# Hydrolysis of Solid Buffer Enables High‐Performance Aqueous Zinc Ion Battery

**DOI:** 10.1002/advs.202307052

**Published:** 2023-12-08

**Authors:** Hao Cheng, Shichao Zhang, Wenxuan Guo, Qian Wu, Zeyu Shen, Linlin Wang, Wei Zhong, Di Li, Bing Zhang, Chengwu Liu, Yewu Wang, Yingying Lu

**Affiliations:** ^1^ State Key Laboratory of Chemical Engineering Institute of Pharmaceutical Engineering College of Chemical and Biological Engineering Zhejiang University Hangzhou 310027 P.R. China; ^2^ ZJU‐Hangzhou Global Scientific and Technological Innovation Center Zhejiang University Hangzhou 311215 P.R. China; ^3^ Institute of Wenzhou Zhejiang University Wenzhou 325006 P.R. China; ^4^ Department of Physics Zhejiang Province Key Laboratory of Quantum Technology, and Device & State Key Laboratory of Silicon Materials Zhejiang University Hangzhou 310027 P.R. China; ^5^ Department of Chemical Engineering Shanghai Jiao Tong University Shanghai 200240 P.R. China

**Keywords:** hydrolysis, interfaces, solid buffers, Zinc ion batteries

## Abstract

Aqueous zinc (Zn) ion batteries (AZIBs) have not yet fulfilled their talent of high safety and low cost since the anode/electrolyte interface (AEI) has long been impeded by hydrogen evolution, surface corrosion, dendritic growth, and by‐product accumulation. Here, the hydrolysis of solid buffers is elaborately proposed to comprehensively and enduringly handle these issues. Take 2D layered black phosphorus (BP) as a hydrolytic subject. It is reported that the phosphoric acid generated by hydrolysis in an aqueous electrolyte produces a zinc phosphate (ZPO) rich solid electrolyte interphase (SEI) layer, which largely inhibits the dendrite growth, surface corrosion, and hydrogen evolution. Meanwhile, the hydrolytic phosphoric acid stabilizes the pH value near AEI, avoiding the accumulation of alkaline by‐products. Notably, compared with the disposable ZPO engineerings of anodic SEI pre‐construction and electrolyte additive, the hydrolysis strategy of BP can realize a dramatically prolonged protective effect. As a result, these multiple merits endow BP modified separator to achieve improved stripping/plating stability toward Zn anode with more than ten times lifespan enhancement in Zn||Zn symmetrical cell. More encouragingly, when coupled with a V_2_O_5_·nH_2_O cathode with ultra‐high loadings (34.1 and 28.7 mg cm^−2^), the cumulative capacities are remarkably promoted for both coin and pouch cells.

## Graphical abstract

Aqueous zinc‐ion batteries (AZIBs) have emerged as a promising energy storage solution because of the virtues of non‐flammability as well as the multiple merits of Zn metal anode. This work presents a solid sustained‐release buffering strategy that continuously reinforces the physicochemical environment of the anode/electrolyte interface, thereby addressing issues such as hydrogen evolution, corrosion, dendrite formation, and by‐products in the AZIBs.

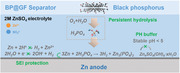

## 1 Introduction

The prevalence of lithium‐ion batteries (LIBs) remains tempered by potential safety concerns, particularly the high flammability associated with their organic electrolytes.^[^ ^1^, ^2^, ^3^ ^]^ Alternatively, aqueous zinc (Zn) ‐ion batteries (AZIBs) have emerged as a promising energy storage solution by the virtues of non‐flammability of aqueous electrolytes as well as the multiple merits of Zn metal anode including, environmental friendliness, high theoretical specific capacity (820 mAh g^−1^) and relatively low redox overpotential (−0.76 V versus standard hydrogen electrode (SHE)).^[^ ^4^, ^5^, ^6^ ^]^ Regrettably, the performance of AZIBs is significantly hindered by various issues affecting the Zn anode, even when using mild electrolytes (e.g., 1–2 m ZnSO_4_ solution). These issues encompass the hydrogen evolution reaction (HER), surface corrosion, dendrite growth, by‐product accumulation, and the development of mutually exacerbating synergies, leading to poor reversibility and shortened lifespan.^[^ ^7^, ^8^, ^9^, ^10^, ^11^ ^]^

To address these obstinate challenges, a plethora of intriguing strategies have been elaborately ^12^, ^13^ ^]^ Among these strategies, modifications to the anode/electrolyte interface (AEI), including Zn anode surface engineering and electrolyte adjustments, have been widely proposed, as the AEI is the primary site of these issues.^[^ ^12^, ^14^, ^15^ ^]^ From the perspective of the Zn anode, the preferential exposure of the thermodynamically favorable (002) crystal plane and the pre‐construction of solid electrolyte interphase (SEI) layer have proven effective in enhancing Zn anode reversibility.^[^ ^5^, ^16^, ^17^, ^18^, ^19^, ^20^ ^]^ However, due to the substantial volume changes experienced by the Zn anode during the stripping/plating process, surface engineering efforts initiated at the outset are inevitably exhausted, as they lack self‐healing capabilities. Therefore, a strategy for continually strengthening the AEI is desirable. In this context, electrolyte modification offers the potential for more durable protection, as it maintains continuous contact with the anodic surface. For example, highly concentrated and/or eutectic electrolytes can continuously interact with the AEI by stabilizing the electric double layer (EDL) or enhancing the SEI layer, thus improving the electrochemical stability of the Zn anode.^[^ ^21^, ^22^, ^23^ ^]^ Nevertheless, the resulting high cost hampers the realization of the inherent advantages of low‐cost AZIBs.

Comparatively, the strategy of adding trace electrolyte additives has garnered significant attention due to its cost‐effectiveness, simplicity, and scalability. To date, various trace additives, including glucose,^[^ ^24^ ^]^ saccharin,^[^ ^15^ ^]^ monosodium glutamate,^[^ ^25^ ^]^ fluorinated surfactant,^[^ ^26^ ^]^ and polymer^[^ ^27^ ^]^ have been widely employed to modulate Zn stripping/plating behavior. These trace additives tend to adsorb and/or decompose on the Zn anode surface, forming an effective EDL or SEI layer, with the aim of inhibiting HER, surface corrosion, dendrite growth, and by‐product accumulation. Impressively, Nazar et al. demonstrated that the introduction of 50 mM *N*,*N*‐dimethylformamidium trifluoromethanesulfonate (DOTf) into 2 m ZnSO_4_‐based aqueous electrolytes can generate a robust SEI layer and significantly enhance the cumulative plated capacity (CPC) of the Zn anode.^[^ ^28^ ^]^ The SEI layer is mainly generated by the electrochemical redox reaction between trace additives and the Zn anode at the initial stage.^[^ ^15^, ^28^ ^]^ This implies that the effectiveness of trace additives diminishes significantly as cycling progresses. On the other hand, the excessive use of trace additives upfront can lead to an undesired overextension of the SEI layer. Therefore, achieving long‐term and consistent effectiveness of trace additives has become an interesting and challenging topic that has barely been involved in previous research.

In this study, we introduce the hydrolysis of a solid buffer as a novel strategy to achieve comprehensive and long‐lasting modulation of the Zn anode. 2D layered black phosphorus (BP) is widely used in the semiconductor field due to its excellent charge carrier mobility, adjustable band gap, and high anisotropy.^[^ ^29^, ^30^, ^31^ ^]^ Furthermore, BP has found application as an anode material for LIBs due to its ultra‐high theoretical capacity (2596 mAh g^−1^) and extremely low energy barrier (0.08 eV).^[^ ^32^, ^33^, ^34^, ^35^ ^]^ However, BP's instability in environments containing oxygen and water has been a major obstacle to its use.^[^ ^36^ ^]^ Fortunately, from another perspective, this “defect” of BP can enable it to serve as a solid buffer in AZIBs due to its slow degradation in water, which releases well‐known phosphoric acid species.^[^ ^37^ ^]^ In our work, we demonstrate that BP can gradually degrade in mild electrolytes, generating phosphoric acid. This allows the BP‐modified glass fiber (BP@GF) separator to form a zinc phosphate (ZPO)‐rich SEI layer on the Zn surface, effectively inhibiting dendrite growth, HER, and surface corrosion. Additionally, the hydrolyzed phosphoric acid stabilizes the pH value of the AEI within a reasonable range, preventing the accumulation of by‐products. Importantly, compared to disposable ZPO engineering for anodic SEI pre‐construction or electrolyte additives, the BP@GF separator significantly improves the reversibility and stability of the Zn anode. We also provide a manufacturing method for cathodes with ultra‐high loadings to fully validate the effectiveness of this strategy. Consequently, we observe a remarkable increase in cumulative capacities for both coin and pouch cells.

## 2 Results and Discussion

**Figure 1:**
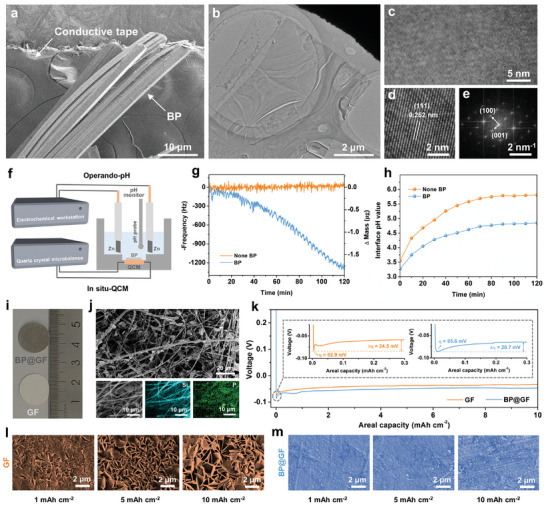
a) SEM image of BP. b) TEM and c) HRTEM images of BP. d) Lattice fringes and e) the corresponding fast flourier transform (FFT) pattern. f) Schematic of the operando‐pH and in situ QCM characterization. The left Zn is stripped and deposited onto the right Zn with a current density of 2 mA cm^−2^. g) Frequency/mass signals and h) in situ pH alterations in f). i) Optical pictures of GF and BP@GF separators. j) SEM images under different magnifications and the corresponding EDS mapping of BP@GF separator. k) Initial plating voltage profiles of Zn||Cu half‐cells with GF and BP@GF separators at 2 mA cm^−2^. Ex situ SEM images of plated Zn on Cu foil with various capacities for l) GF and m) BP@GF separators.

2D layered black phosphorus (BP) was synthesized using a chemical vapor transport method, as detailed in the experimental section (Supporting Information). The scanning electron microscope (SEM) image presented in **Figure** **1**a illustrates the bulk BP in the form of stacked layers. Subsequent ultrasonic exfoliation led to the formation of large‐scale, few‐layered BP, as revealed by the transmission electron microscope (TEM) image in Figure 1b. This structural transformation aligns with the typical characteristics of 2D layered materials. The high‐resolution TEM image, as shown in Figure 1c, highlights a wide range of well‐ordered crystal plane arrangements, underscoring the excellent crystallinity of BP. An enlarged view (Figure 1d) displays clear lattice fringes with a d‐spacing of 0.252 nm, matching the (111) plane of BP. Furthermore, the corresponding fast Fourier transformation (FFT) pattern (Figure 1e) provides further confirmation of the high crystallinity.^[^ ^38^ ^]^ Additionally, the X‐ray diffraction (XRD) pattern, Raman spectrum, and X‐ray photoelectron spectroscopy (XPS) profile, as depicted in Figure S1 (Supporting Information), consistently support the exceptional quality of the synthesized BP.

A custom‐designed operando‐pH and in situ quartz crystal microbalance (QCM) system (Figure S2, Supporting Information) was employed in conjunction to investigate the hydrolysis behavior of BP when used with a practical 2 m ZnSO_4_ electrolyte in a Zn||Zn symmetric cell, as schematically illustrated in Figure 1f. The mass information, derived from frequency alterations, reveals a gradual reduction in BP mass during the electroplating process, indicating the hydrolysis of BP (Figure 1g). Additional evidence of BP's long‐term hydrolysis property in the 2 m ZnSO_4_ electrolyte is provided by ion chromatography (Figure S3, Supporting Information) and ex situ QCM results (Figure S4, Supporting Information). It is widely recognized that the pH value in the vicinity of the anode/electrolyte interface (AEI) significantly influences the electrochemical reversibility of the Zn anode.^[^ ^7^ ^]^ Due to the hydrogen evolution reaction (HER) in aqueous electrolytes, the pH value at the interface tends to gradually increase, resulting in the accumulation of by‐products such as alkaline zincate (Zn_x_SO_y_(OH)_z_·nH_2_O, ZHS). These by‐products can lead to the passivation of the AEI.^[^ ^39^, ^40^ ^]^ Notably, during the electrodeposition process (Figure 1h), the pH value surrounding the AEI, where BP is present, undergoes a slight increase from 3.25 and then stabilizes at ≈4.85. In stark contrast, the pH value undergoes significant changes in the absence of BP, reaching a high value of 5.81 in the baseline. These results demonstrate that BP undergoes hydrolysis in the electrolyte, effectively buffering the pH around the AEI.

Confirming the hydrolysis and pH‐buffer behaviors of BP in electrolytes, we incorporated BP into a GF separator through a straightforward ultrasonic dispersion method to modulate the anode/electrolyte interface (AEI) of Zn anode in practical cells. As depicted in Figure 1i, the BP@GF separator undergoes a noticeable change from white to dark grey. SEM images, energy dispersive spectrometer (EDS) mapping of BP@GF (Figure 1j), and the pristine morphology of GF (Figure S5, Supporting Information) collectively demonstrate the uniform distribution of BP on the GF separator.

A Zn||Cu half‐cell was assembled to preliminarily assess the nucleation and growth behavior of Zn in the presence of BP. Figure 1k illustrates the voltage profiles during the Zn electro‐crystallization process in 2 m ZnSO_4_ electrolytes with GF and BP@GF separators. Notably, the BP@GF separator exhibits a somewhat blunter and slightly larger sharp nucleation overpotential (η) of 85.6 mV when compared to the baseline GF separator (82.9 mV). This increase in interface impedance is speculated to be linked to the generation of the solid electrolyte interphase (SEI) layer. Conversely, the growth overpotential (△η) is reduced to 20.7 mV in the BP@GF cell, a value lower than that of the GF cell (24.5 mV). Reduced growth overpotential is widely recognized as advantageous for depositing Zn as smaller crystals rather than large dendrites.^[^ ^28^ ^]^ To further validate this, the morphological and structural evolution of Zn electro‐crystallization was examined through ex situ SEM observations, with 1, 5, and 10 mAh cm^−2^ of Zn being deposited on a Cu substrate. As shown in Figure 1l, the GF separator results in the formation of random dendrites and hexagonal nanosheets (confirmed as ZHS, as illustrated in Figure S6, Supporting Information). These structures gradually grow with increasing deposition capacity. In contrast, when using the BP@GF separator, Zn is deposited with a robust and flat structure, and dendrites and by‐products are rare, even at high deposition capacities (Figure 1m). Additionally, electrochemically in situ optical photos (Figure S7, Supporting Information) further corroborate these findings. These results underscore the inhibitory effect of BP hydrolysis on dendrite growth and by‐product accumulation during the Zn deposition process.

**Figure 2:**
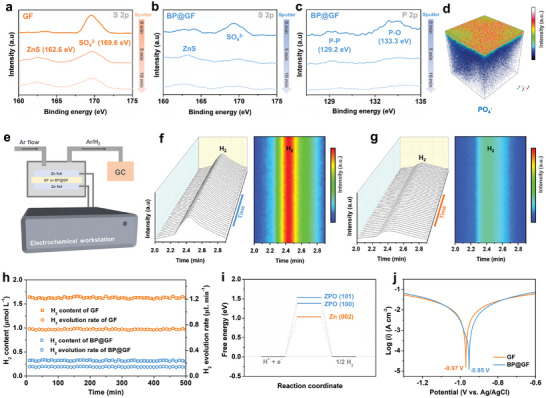
XPS spectra of S‐2p and P‐2p orbits for the platted Zn (10 mAh cm^−2^) in the Zn||Cu half‐cells with a) GF and b,c) BP@GF separators. d) 3D views of the PO_4_^−^ signals by TOF‐SIMS. e) Schematic of the in situ electrochemical GC device for quantitative detection of hydrogen evolution. Time‐resolved EC‐GC profiles and the corresponding contour maps of Zn||Zn symmetrical cells with f) GF and g) BP@GF separators at 5 mA cm^−2^. h) Quantitative results of hydrogen evolution. i) Calculated HER free energy diagram of Zn (002), ZPO (100), and (101). j) Tafel curves of symmetric cell configurations with GF and BP@GF separators.

To understand the improvements at the interface, we examined the properties of the solid electrolyte interphase (SEI). As depicted in **Figure** **2**a, a strong XPS signal peak for SO_4_^2−^ is observed on the surface of Zn plated to 10 mAh cm^−2^ in the GF cell. Even after 10 minutes of sputtering, the interior still exhibits a high‐intensity SO_4_^2−^ signal, indicating the presence of a relatively thick ZHS layer. The formation of internal zinc sulfide can be attributed to the reduction of sulfur species in the electrolyte, which contributes to the composition of the SEI. In contrast, the BP@GF cell also exhibits a strong SO_4_^2−^ signal (Figure 2b), likely originating from surface ion adsorption. However, with the sputtering process, the internal SO_4_^2−^ signal becomes extremely weak, confirming the absence of alkaline ZHS by‐products.

On the other hand, XPS results for the P 2p orbital reveal the presence of abundant P‐P and P‐O signals on the surface of the Zn plated in the BP@GF cell (Figure 2c), suggesting the formation of a P‐containing SEI layer. Further validation is provided by the time‐of‐flight secondary ion mass spectrometry (TOF‐SIMS) result (Figure 2d), which confirms the predominant presence of zinc phosphate (Zn_3_(PO_4_)_2_, ZPO) on the Zn surface, accompanied by a small amount of other phosphates (Figure S8, Supporting Information).

To further assess the benefits of the ZPO‐rich SEI, in situ electrochemical gas chromatography (GC) was employed for quantitative evaluation of the hydrogen evolution reaction (HER). The Zn foil was used as both the working and counter electrode (Figure 2e) and underwent reversible Zn plating/stripping at a current density of 5 mA cm^−2^. A constant flow of argon (25 sccm, standard cubic centimeters per minute) was introduced to channel the generated hydrogen into the GC. The standard H_2_ peak is observed at ≈2.45 min (Figure S9, Supporting Information).

Here, we present a theoretical derivation model for the hydrogen evolution reaction (HER) rate. Let's define the volume of the container as *V* (20 mL, as shown in Figure S10, Supporting Information), the flow rate of argon as *x* (sccm), the HER rate as *y* (sccm), and the proportion (expressed as a percentage) of argon at time *t* as *ρ_x_(t)*, and the proportion of hydrogen as *ρ_y_(t)*. At time *t* + Δ*t*, where Δ*t* represents a small change in time, the hydrogen content satisfies the following equation:

(1) $$V\cdot \rho_{y}\left(t+\Delta t\right)=V\cdot \rho_{y}\left(t\right)+y\cdot \Delta t-\left(x+y\right)\cdot \Delta t\cdot \rho_{y}\left(t\right)$$

With terms rearrangement, we have

(2) $$\frac{\rho_{y}\left(t+\Delta t\right)-\rho_{y}\left(t\right)}{\Delta t}=\frac{y}{V}-\frac{x+y}{V}\rho_{y}\left(t\right)$$

Further, the derivative of ρ_*y*_(*t*) with respect to time *t* can be calculated as:

(3) $${\dot{\rho }}_{y}\left(t\right)=\lim_{\Delta t\to 0}\frac{\rho_{y}\left(t+\Delta t\right)-\rho_{y}\left(t\right)}{\Delta t}=\frac{y}{V}-\frac{x+y}{V}\rho_{y}\left(t\right)$$

Finally, we get the differential equation that the concentration of hydrogen satisfies as:

(4) $$\begin{array}{c} \left\{{\dot{\rho }}_{y}\left(t\right)=\frac{y}{V}-\frac{x+y}{V}\rho_{y}\left(t\right)\right.\\ \rho_{y}\left(0\right)=0 \end{array}$$

Solving this ordinary differential equation, we get the explicit expression of the concentration of hydrogen:

(5) $$\rho_{y}\left(t\right)=\frac{y}{x+y}-\frac{y}{x+y}\cdot e^{-\frac{x+y}{V}t}$$

According to the practical conditions, the steady‐state concentration ratio of hydrogen will be $\frac{y}{x+y}$, while only taking a few minutes.

As illustrated in Figure 2f, once H_2_ release reaches a steady state, distinct H_2_ signals become detectable in the GF cell, maintaining a corresponding H_2_ concentration of 1.64 µmol L^−1^ (Figure 2h). Conversely, the H_2_ release in the cell with BP@GF is significantly attenuated (Figure 2g), resulting in an H_2_ concentration of only 0.35 µmol L^−1^ (Figure 2h). Through quantitative calculation, we determined the H_2_ release rates as 0.74 and 0.14 µL min^−1^ for GF and BP@GF cells, respectively (Figure 2h). This clearly demonstrates the suppression of H_2_ evolution facilitated by BP hydrolysis. It is reasonable to speculate that this inhibitory effect is closely related to the ZPO‐rich SEI layer. Unambiguous confirmation of this deduction comes from the significantly inhibited hydrogen evolution observed in the ZPO@Zn assembled cell (Figure S11, Supporting Information). Furthermore, to provide in‐depth theoretical verification, density functional theory (DFT) calculations were performed (Figure S12, Supporting Information). It is well‐known that the (002) plane of Zn is thermodynamically unfavorable for the hydrogen evolution reaction (HER), possessing a higher free energy for HER.^[^ ^16^ ^]^ As depicted in Figure 2i, the higher HER free energies corresponding to the typical low‐index planes of ZPO (100) and (101), measuring 1.39 and 1.53 eV respectively, indicate that the ZPO‐rich SEI layer can indeed inhibit HER. Additionally, based on the Tafel curves in Figure 2j, the corrosion potential of the BP@GF cell shifts to a higher value (−0.95 V) compared to that of the GF cell (−0.97 V), signifying effective suppression of anodic corrosion by the ZPO‐rich SEI layer. It's worth noting that while the formation of the ZPO layer can generate a small amount of H_2_ through a chemical reaction, it is evident that the ZPO layer can efficiently electrochemically suppress H_2_ production and dominate the process.

**Figure 3:**
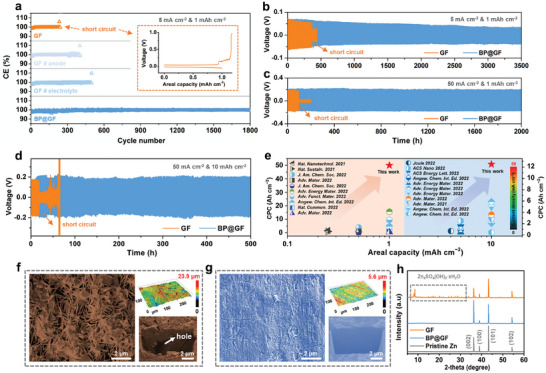
a) CE tests of Zn||Cu half‐cells with GF, GF#anode, GF#electrolyte, and BP@GF at 5 mA cm^−2^ and 1 mAh cm^−2^. GF, GF#anode, GF#electrolyte and BP@GF represent Zn||Cu half‐cell with GF separator and 2 m ZnSO_4_, ZPO@Zn||Cu half‐cell with GF separator and 2 m ZnSO_4_, Zn||Cu half‐cell with GF separator and 20 mM H_3_PO_4_/2 m ZnSO_4_, and Zn||Cu half‐cell with BP@GF separator and 2 m ZnSO_4_, respectively. The inset is the GCD curve in GF case. Time–voltage curves of Zn||Zn symmetrical cells with GF and BP@GF separators at b) 5 mA cm^−2^ and 1 mAh cm^−2^, c) 50 mA cm^−2^ and 1 mAh cm^−2^ and d) 50 mA cm^−2^ and 10 mAh cm^−2^. e) Comparison of the CPC and areal capacity between our work and very recent reports. Top‐view SEM mages (left), 3D confocal images (top right), and cross‐sectional images produced by FIB (lower right) of cycled Zn (100 h) in the Zn||Zn symmetrical cells with f) GF and g) BP@GF separators. h) XRD patterns of pristine Zn and cycled Zn (100 h) in the Zn||Zn symmetrical cells with GF and BP@GF separators.

Coulombic efficiency (CE) tests were conducted in Zn||Cu half‐cells to assess the consecutive Zn plating/stripping behavior and utilization at current densities of 5 mA cm^−2^ and 1 mAh cm^−2^. As depicted in **Figure** **3**a, the CE of the BP@GF cell quickly exceeds 99% within the initial 20 cycles and stabilizes at 99.5% over 1800 cycles, with stable galvanostatic charge‐discharge (GCD) curves (Figure S13, Supporting Information). In the case of the GF cell, although CE can also be maintained at a high level (99.5%), it experiences a sudden sharp rise at the 287th cycle, which is attributed to a soft short circuit in the battery, accompanied by a disrupted charging curve (inset). It's noteworthy that while the pre‐construction of the ZPO SEI (Figures S14–S17, Supporting Information) and the use of trace H_3_PO_4_ additive with a GF separator can maintain highly stable CE values (both exceeding 99.5%), their reversible circularities fall significantly short of the BP@GF cell. This underscores the advantages of the continuous interface reinforcement strategy achieved through the hydrolysis of BP.

Zn||Zn symmetrical cells were assembled to further evaluate the Zn anode's stripping/plating behavior. Impressively, the Zn||Zn cell with the BP@GF separator demonstrates outstanding stability, maintaining steady cycling for up to 3500 hours at 5 mA cm^−2^ and 1 mAh cm^−2^ (Figure 3b). The BP@GF separator enhances the Zn||Zn cell's cycle life compared to the GF cell, as well as cells with pre‐formed ZPO SEI or those with added trace H_3_PO_4_ (Figure S18, Supporting Information). Remarkably, even at a high current density of 50 mA cm^−2^, the BP@GF cell exhibits stable stripping/plating behavior for over 2000 hours (Figure 3c), while the GF cell can only cycle for about 100 hours before an open circuit occurs. Figure S19 (Supporting Information) shows that after 100 hours of cycling, the coin cell has significantly expanded in thickness, indicating rapid internal pressure buildup due to severe HER. Notably, when the areal capacity increases to a high value of 10 mAh cm^−2^, the cycle time can still be maintained for over 500 hours in the BP@GF cell (Figure 3d). Cumulative plated capacity (CPC) is commonly used to quantitatively evaluate the stability of the Zn anode. Under areal capacities of 1 and 10 mAh cm^−2^, the corresponding CPCs reach 50.0 and 12.5 Ah cm^−2^, respectively. These values surpass those reported in most recent works, highlighting the advantage of the BP hydrolysis strategy (Figure 3e and Table S1, Supporting Information).

SEM analysis was conducted to examine the morphology of the Zn anode after 100 hours of cycling. As depicted in Figure 3f, in the GF cell, the Zn surface is covered with a multitude of sheet‐like by‐products as a result of the continuous stripping/plating process. Top‐view (Figure S20, Supporting Information) and 3D confocal images (top right of Figure 3f) further reveal an inhomogeneous surface structure with a height fluctuation as high as 23.9 µm. The focused ion beam (FIB) SEM image (lower right of Figure 3f) highlights the uneven surface structure and interior holes. In stark contrast, the surface of the Zn anode in the BP@GF cell displays significant improvement in flatness (Figure 3g and Figure S20, Supporting Information), with no obvious sheet‐like products observed. Surface undulations are notably reduced to just 5.6 µm, and the interior Zn structure becomes more compact, confirming the unambiguous electrochemical process. Further comparisons in Figures S21 and S22 (Supporting Information) demonstrate that the morphology of the Zn anode using the BP@GF separator is more uniform compared to pre‐formed ZPO SEI and the addition of trace H_3_PO_4_, even at longer cycle times (300 h).

The XRD pattern (Figure 3h) reveals that, after cycling for 100 hours, a series of additional peaks emerge alongside the characteristic peak of Zn metal. These extra peaks correspond to the Zn_4_SO_4_(OH)_δ_·xH_2_O by‐product, which is consistent with the SEM results (Figure 3f) and prior findings.^[^ ^7^, ^41^ ^]^ Conversely, the BP@GF cell shows minimal by‐product signals. As mentioned earlier, the preferred orientation of the (002) plane is conducive to inhibiting HER and dendrite formation. Consequently, the intensity of the (002) plane of the Zn anode in the BP@GF cell is significantly higher than that of the GF case and even stronger than that of pristine Zn metal. This indicates that, in addition to the SEI layer, the stripping/plating process facilitates the reconstruction of the Zn crystal configuration, enhancing its electrochemical stability and supporting an extended lifespan with an ultra‐high CPC.

**Figure 4:**
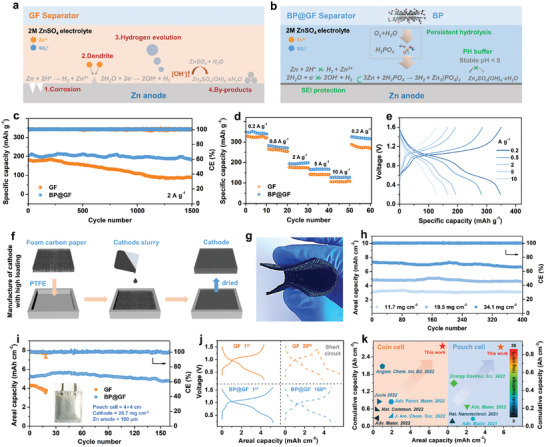
Schematic diagram of a) challenges for Zn anode and b) mechanisms of BP@GF separator for improving the Zn issues. c) Cycle and d) Rate performances of Zn||V_2_O_5_·nH_2_O cells with GF and BP@GF separators. The mass loading of V_2_O_5_·nH_2_O is ≈1.5 mg cm^−2^. e) GCD curves of Zn||V_2_O_5_·nH_2_O cell with BP@GF separator in d). f) Schematic illustration of the manufacture of cathode with high mass loadings. g) Photograph of V_2_O_5_·nH_2_O@FCP cathode, highlighting the flexibility and integrity in the bending state. h) Cycle performances of Zn||V_2_O_5_·nH_2_O@FCP cells with BP@GF separators at various high mass loadings. i) Cycle performances of Zn||V_2_O_5_·nH_2_O@FCP pouch cells with GF and BP@GF separators. j) The corresponding GCD curves of cells in i). k) Comparison of the cumulative capacity and areal capacity for the coin and pouch cells between our work and very recent reports.

The beneficial effects of BP hydrolysis are summarized in **Figure** **4**a,b. The in situ generation of phosphoric acid through BP hydrolysis in the electrolyte contributes to several advantages. First, it results in the formation of a uniform, ZPO‐rich SEI layer on the Zn anode, effectively preventing chemical corrosion between metallic Zn and the mildly acidic electrolyte. Second, this in situ SEI layer aids in the control of the AEI, inhibiting dendrite growth. Thirdly, the passivated ZPO‐rich SEI layer suppresses the HER process. Fourthly, the in situ hydrolyzed phosphoric acid helps maintain a stable pH level near the AEI, preventing the excessive rise in pH and thus greatly reducing by‐product accumulation. Lastly, this continuous and in situ hydrolysis ensures the durability of these benefits, achieving ultra‐long electrochemical stability.

To comprehensively evaluate the performance of the BP@GF separator, full cells were assembled with V_2_O_5_·nH_2_O as the cathode material. V_2_O_5_·nH_2_O powder was synthesized using a hydrothermal method,^[^ ^42^ ^]^ and its structure and morphology are depicted in Figure S23 (Supporting Information). V_2_O_5_·nH_2_O was deposited onto carbon paper with a mass loading of ≈1.5 mg cm^−2^. As depicted in Figure 4c, the Zn||V_2_O_5_·nH_2_O cell with a BP@GF separator (referred to as BP@GF cell) exhibits superior cyclic stability. Initially, the BP@GF cell achieves a specific capacity as high as 203 mAh g^−1^ at 2 A g^−1^. Even after 2000 cycles, the reversible capacity remains at 182 mAh g^−1^, with a capacity retention of 90%. In contrast, the GF cell exhibits a decay in reversible capacity, dropping to only 99 mAh g^−1^ due to the previously mentioned issues with the Zn anode. Furthermore, the BP@GF cell demonstrates excellent rate performance, delivering reversible capacities of 348, 282, 194, 166, and 127 mAh g^−1^ at 0.2, 0.5, 2, 5, and 10 A g^−1^, respectively, surpassing the performance of the GF cell (Figure 4d). Stable GCD curves at various current densities confirm the highly reversible electrochemical reaction process (Figure 4e), consistent with cyclic voltammetry (CV) results (Figure S24, Supporting Information).

The performance observed under low cathode loading effectively validates the effectiveness of the BP@GF separator. However, AZIBs ultimately need to operate under harsh conditions, particularly with high areal capacity, which entails high cathode loading. Currently, manufacturing large‐scale cathodes with ultra‐high loading faces challenges because thick metal oxide‐based cathodes are prone to cracking when applied to conventional current collectors (e.g., titanium foil and carbon paper, Figure S25, Supporting Information). To address this, a flexible and lightweight foam carbon paper (FCP, Figure S26, Supporting Information) is used as the substrate to produce large‐sized, high‐loading cathodes through a simple and scalable casting method (Figure 4f and Figure S27, Supporting Information). As shown in Figure 4g, the V_2_O_5_·nH_2_O@FCP cathode, with an active mass loading of 34.1 mg cm^−2^, maintains its structural integrity and flexibility. The Zn||V_2_O_5_·nH_2_O@FCP cell with a BP@GF separator achieves an initial areal capacity of 3.1 and 4.8 mAh cm^−2^ at 0.5 A g^−1^ with active mass loadings of 11.7 and 19.5 mg cm^−2^, resulting in capacity retentions of up to 98.7% and 95.6%, respectively (Figure 4h). Even under an ultra‐high loading of 34.1 mg cm^−2^, a high capacity retention of 90.7% is maintained, with a reversible areal capacity of 6.68 mAh cm^−2^ after 400 cycles at 0.5 A g^−1^. The cycling stability of a 4×4 cm pouch cell with a mass loading of 28.7 mg cm^−2^ is also assessed (inset of Figure 4i and Figure S28, Supporting Information). As shown in Figure 4i, the initial areal capacity of the BP@GF cell is 5.3 mAh cm^−2^ at 0.5 A g^−1^, surpassing that of the GF separator (4.3 mAh cm^−2^). After 160 cycles, the BP@GF cell maintains 90.1% of its capacity, with well‐defined GCD curves (Figure 4j). For the GF battery, during the 20^th^ cycle of the charging process, a sharp voltage drop is observed when the battery is charged to 1.43 V, indicating a short‐circuit event in the battery. Impressively, the cumulative capacities reach 2.8 and 0.88 Ah cm^−2^ for the coin and pouch cells. As shown in Figure 4k and Table S2 (Supporting Information), compared to recent works in this field, our approach demonstrates superior performance, highlighting the effectiveness of our comprehensive strategy. Figure S29 (Supporting Information) illustrates that the pouch cell can continuously power a timer with a 1.5 V input even after folding and partial cutting, demonstrating its durability and safety.

## 3 Conclusion

In summary, we have introduced a solid buffer that offers a comprehensive and long‐lasting solution to address the challenges associated with AZIBs. Our research demonstrates that the continuous hydrolysis of 2D layered BP, resulting in the production of phosphoric acid species in a mild electrolyte environment, confers several key advantages. First, the phosphoric acid species effectively lower the pH value near the AEI, preventing the formation of alkaline ZnSO_4_(OH)_δ_·xH_2_O byproducts. Second, the in situ formation of a zinc phosphate SEI layer on the Zn surface reduces the growth overpotential, facilitating dendrite‐free Zn deposition. Thirdly, both experimental and theoretical results demonstrate that the zinc phosphate SEI layer significantly inhibits the side reactions of HER and corrosion. Lastly, the continuous hydrolysis of BP ensures that these benefits persist over time. Consequently, this comprehensive and sustained modification of the separator with BP leads to substantially improved stripping/plating stability. This improvement is reflected in a high cumulative plated capacity of up to 50 Ah cm^−2^ for Zn||Zn symmetrical cells and remarkable cycle life with a cumulative capacity of up to 0.88 Ah cm^−2^ for pouch cells, even with a high cathode mass loading of up to 28.7 mg cm^−2^. Our findings provide a novel approach for achieving the high stability of Zn anodes while addressing multiple challenges, thereby paving the way for future commercialization.

## Conflict of Interest

The authors declare no conflict of interest.

## Supporting information

Supporting Information

## Data Availability

The data that support the findings of this study are available from the corresponding author upon reasonable request.
