# Supplementary material for: Hydrolysis of Solid Buffer Enables High‐Performance Aqueous Zinc Ion Battery
Source: Adv Sci (Weinh). 2023 Dec 8;11(7):2307052. doi: 10.1002/advs.202307052 (PMC10870042; doi:10.1002/advs.202307052)
Supplement: Supplementary file 1 — Supporting Information [file ADVS-11-2307052-s001.pdf]

ADVANCED SCIENCE
Open Access

Supporting Information

for *Adv. Sci.*, DOI 10.1002/advs.202307052

Hydrolysis of Solid Buffer Enables High-Performance Aqueous Zinc Ion Battery

*Hao Cheng, Shichao Zhang, Wenxuan Guo, Qian Wu, Zeyu Shen, Linlin Wang, Wei Zhong, Di Li, Bing Zhang, Chengwu Liu, Yewu Wang and Yingying Lu**

Hydrolysis of solid buffer enables high performance aqueous zinc ion battery

Hao Cheng [a,b,c], Shichao Zhang [a], Wenxuan Guo [d], Qian Wu [a,b], Zeyu Shen [a,b], Linlin Wang [b], Wei Zhong [a,c], Di Li [a,b], Bing Zhang [a,b], Chengwu Liu [e], Yewu Wang [d], Yingying Lu [a,b,c*]

a. State Key Laboratory of Chemical Engineering, Institute of Pharmaceutical Engineering, College of Chemical and Biological Engineering, Zhejiang University, Hangzhou 310027, China.

b. ZJU-Hangzhou Global Scientific and Technological Innovation Center, Zhejiang University, Hangzhou 311215, China.

c. Institute of Wenzhou, Zhejiang University, Wenzhou 325006, China.

d. Department of Physics, Zhejiang Province Key Laboratory of Quantum Technology and Device & State Key Laboratory of Silicon Materials, Zhejiang University, Hangzhou 310027, China.

e. Department of Chemical Engineering, Shanghai Jiao Tong University, Shanghai 200240, P.R. China.

[*] Corresponding author. *E-mail*: yingyinglu@zju.edu.cn (Yingying Lu)

Experiments

Synthesis of BP

Red phosphorus (500 mg, Aladdin, 99.999% metal basis), iodine (10 mg, Alfa Aesar, 99.999% trace metal basis), and lead (18 mg, Aladdin, powder, 99.95% metal basis) were mixed and transferred into a quartz ampule in an argon-filled glove box. The ampule was sealed and then placed horizontally into a muffle furnace. Subsequently, the furnace was heated to 873 K at a rate of 2 K min^{-1}, kept at this temperature for 2 hours, cooled to 738 K after 490 min, maintained for another 2 hours, and finally cooled down to room temperature naturally.

Preparation of BP@GF

A certain amount of BP (0.5 g) was added into the N-methylpyrrolidone (NMP) solvent (50 mL). The mixture was ultrasonically treated for 1 hour. Subsequently, the purchased GF separators were added to the mixture and ultrasonicated for 20 minutes. In this work, the GF separators were purchased from Whatman including GF/A (260 μm in thickness, 5.2 mg cm^{-2}) and GF/D (675 μm in thickness, 11.5 mg cm^{-2}). The BP-loaded GF (BP@GF) separators were dried in a vacuum oven at 60°C for 12 hours. Then the separators were cut into disks with a diameter of 16 mm or square pieces (45*45 mm). The mass loadings of BP on GF/A and GF/D were 1.78 mg and 2.32 mg cm^{-2}, respectively.

Preparation of electrodes

Phosphoric acid aqueous solution (H_3PO_4, 85 wt.%) and zinc sulfate ($ZnSO_4 \cdot 7H_2O$) powder used in the experiment were purchased from Aladdin. Zn foil (>99.9%) with a thickness of 100 μm was used as the anode. The ZPO@Zn anode was prepared by immersing the Zn foil into the 20 mM H_3PO_4 for 2 hours. The $V_2O_5 \cdot nH_2O$ cathode was synthesized by the reaction of V_2O_5 powder with H_2O_2 at room temperature. V_2O_5 powder (3.64 g, Aladdin) and 30% H_2O_2 (16 mL, Aladdin) were added into 200 mL of deionized water. After aging for 24 hours, the obtained $V_2O_5 \cdot nH_2O$ sediment was collected, washed and freeze-dried for 12 h. The $V_2O_5 \cdot nH_2O$ powder was mixed with polyvinylidene fluoride (PVDF) with a mass ratio of 9:1. The mixture was then dispersed in NMP solvent. For the low-mass loading cathode, the

slurry was cast on the carbon paper. For the high-loading cathode, the slurry was casted into the polytetrafluoroethylene groove that was filled with foam carbon paper (FCP). The FCP used in this work has a self-mass loading of 16.5 mg cm^{-2}, with a thickness of 2 mm, and it can be customized as per requirements. Then, the electrode and groove were dried in a vacuum oven at 60°C for 12 hours. The obtained electrodes were cut into disks with a diameter of 12 mm or square pieces (40*40 mm). The cathodes with different loadings (11.7, 19.5 and 34.1 mg cm^{-2}) were obtained by adjusting the ingestion of slurry. The actual capacity of Zn (100 μm) is 58.5 mAh cm^{-2}, [1] corresponding to a negative/positive (N/P) ratio of 11 in pouch cell.

Battery assembly and electrochemical measurements

The 2025-type button cell was used for electrochemical performance analysis. Zn or ZPO@Zn were used as an anode. $V_2O_5{\cdot}nH_2O$ based electrode with different mass loadings were used as cathodes. GF (GF/A and GF/D) or BP@GF was used as a separator. 2 M $ZnSO_4$ or 20 mM H_3PO_4/2 M $ZnSO_4$ aqueous solution was applied as electrolytes. In the Zn||Cu half cell and Zn||Zn symmetrical cell, the amount of electrolyte was 50 and 100 μL for GF/A or GF/D based separators. In this work, GF/A based membrane was used as a separator when the areal capacity was less than 2 mAh cm^{-2}, and GF/D based membrane was used as a separator when the deposition surface capacity was more than 2 mAh cm^{-2}. The galvanostatic charge-discharge (GCD) tests were performed on a Land system. Electrochemical impedance spectroscopy (EIS), cyclic voltammetry (CV) and Tafel curves were performed on an electrochemical workstation (Chenhua, chi760d). The Tafel test was conducted by scanning from -0.6 to -1.3 V.

Materials characterizations

The scanning electron microscopy (SEM, SIRION-100) equipped with an energy dispersive spectrometer (EDS, Oxford) attachment and the transmission electron microscopy (TEM, JEM-2100) were applied to detect the morphology and structure. The contact angle measurements were performed in a Dataphysics OCA20 system. The operando-pH and in situ QCM characterization were performed by a home-made system including an electrochemical workstation (Chenhua, 760), an electrochemical

quartz crystal microbalance (Chenhua, CHI-440) and a pH probe. The crystal structure was identified by X-ray diffractometer (XRD, SHIMADZU-7000). Raman test was carried out on a Horiba JY LabRAM HR Evolution instrument (532 nm). The surface morphology of Zn anode was observed by a laser confocal microscope (KEYENCE, VK-X150). The cross section of Zn anode was analyzed by dual-beam SEM (FEI Strata 400S). Ion information was detected on the ion chromatography. (Thermo Scientific ICS-5000+). In-situ optical tests were realized under an optical microscope (Giorgione). The X-ray photoelectron spectroscopy (XPS, Escalab 250Xi) with Al Kα radiation was used to identify the surface chemistry. The surface and depth distributions of Zn anode were identified by a time-of-flight secondary ion mass spectrometry (TOF-SIMS, 5-100/ION). The sputtering and analysis areas were 250×250 and 50×50 μm^2, respectively. In situ time-resolved GC test was realized in a breather valve with a volume of 20 mL, in which a 2025-type button cell was placed. The Φ2 mm hole was opened in negative shell to release hydrogen. Argon was used as carrier with a flow rate of 25 sccm (standard cubic centimeter per minute).

DFT calculations

The density functional theory (DFT) calculations were performed with the CASTEP package in Materials Studio. Generalized gradient approximation (GGA) with the Perdew–Burke–Ernzerhof (PBE) functional was selected to describe the electronic exchange correlation energy.[2] The Zn (002), ZPO (101) and ZPO (100) surfaces were modeled as the substrates. The thickness of vacuum layer was set as 15 Å. The Kohn–sham orbits on the basis of plane wave was expanded with a cut-off energy of 600 eV. For geometry optimization, the energy and force convergence were less than 10^{-5} eV and 0.05 eV $Å^{-1}$, respectively.

The Gibbs free-energy (ΔG_{H^*}) was defined as: $\Delta G_{H^*} = \Delta E_{H^*} + \Delta E_{ZPE} - T\Delta S$, where ΔE_{H^*} represents the adsorption energy of hydrogen species on the substrate surface, ΔE_{ZPE} represents the zero-point energy difference of H in adsorbed state and gas phase state. ΔS represents the entropy change of H^* adsorption. Due to the entropy of hydrogen in absorbed state was negligible, ΔS can be calculated as -1/2 S_0, where S_0 was the entropy of H_2 in the gas phase at standard conditions. Therefore, the free energy

of the adsorbed state can be simplified as: $\Delta G_{H*} = \Delta E_{H*} + 0.24\ eV$.[3] The H adsorption energy was defined as:

$$\Delta E_{ads} = 1/n\ [E(sub+nH) - E(sub) - n/2\ E(H_2)]$$

where n was the number of H atoms in the model, *E(sub+nH)* was the total energy of H adsorbed molecules, *E(sub)* was the energy of substrate and $E(H_2)$ was the energy of isolated molecule.

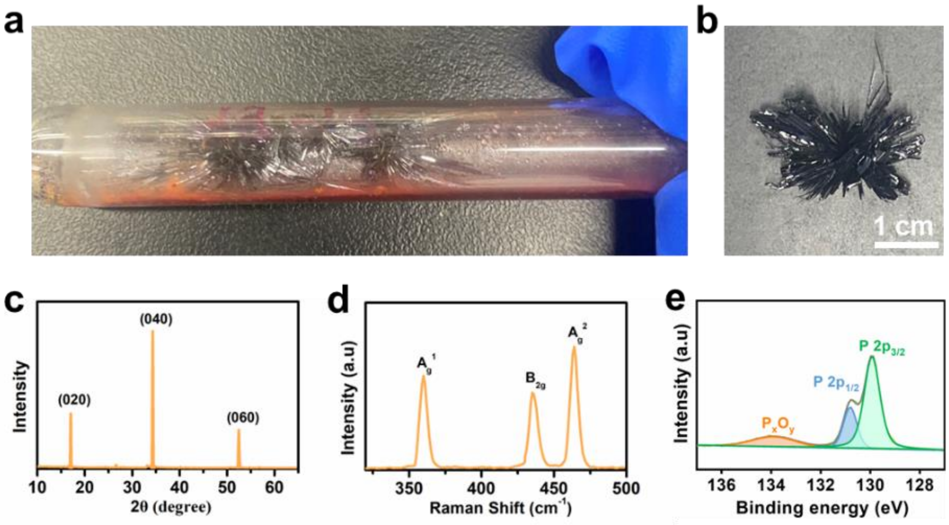

Fig. S1 (a) Calcined quartz ampoules. (b) Optical image of BP. (c) XRD, (d) Raman and (e) XPS result of BP.

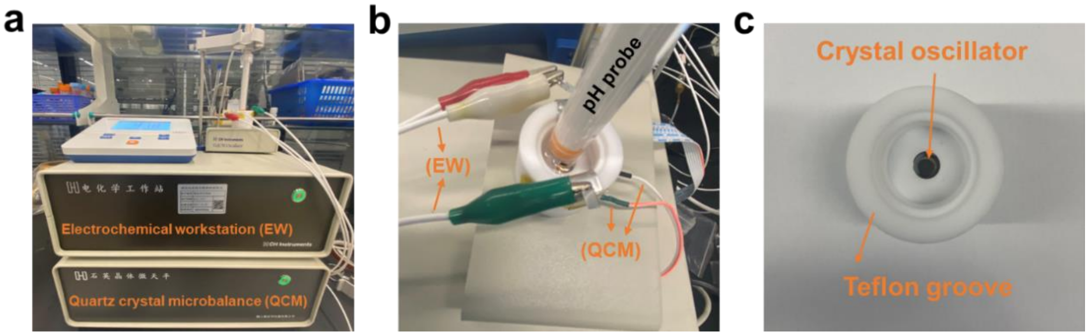

Fig. S2 The home-made operando-pH and in situ QCM system.

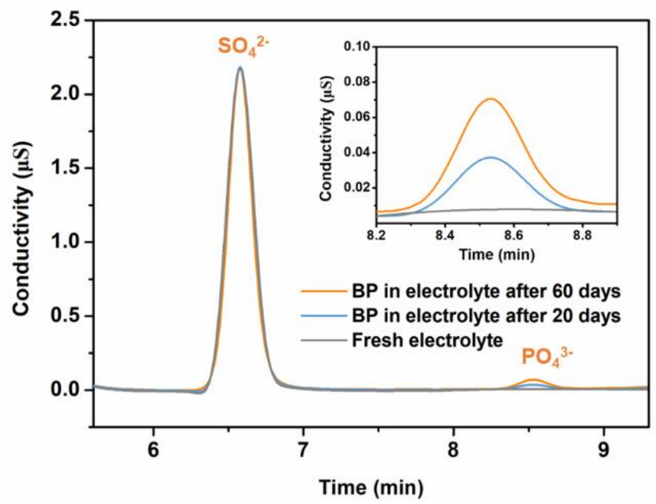

Fig. S3 Hydrolysis of BP revealed by ion chromatography in 2M $ZnSO_4$ electrolyte.

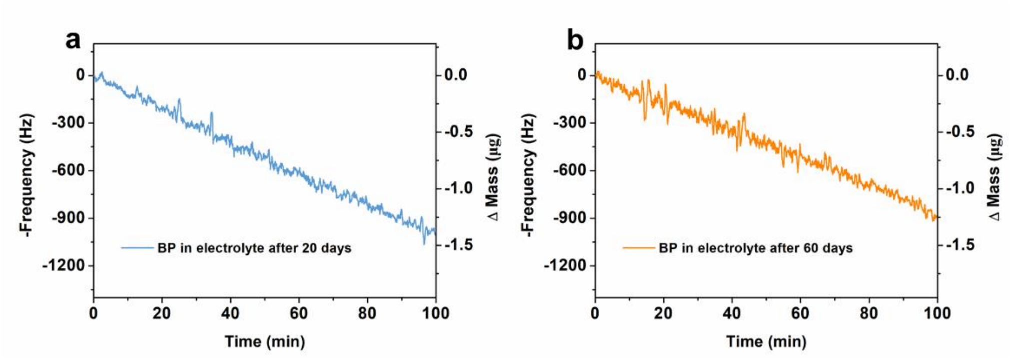

Fig. S4 Ex situ QCM results of BP in 2M $ZnSO_4$ electrolyte after aging (a) 20 and (b) 60 days.

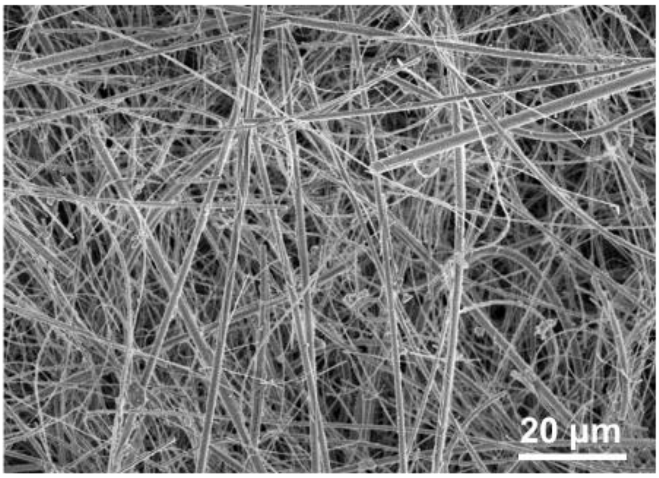

Fig. S5 SEM image of GF separator.

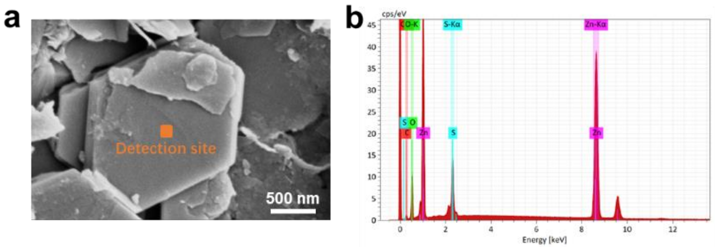

c

Element	Atomic number	Atomic content (a.u.)
O	8	55.9%
S	16	7.6%
Zn	30	36.5%

Fig. S6 (a) SEM image of the hexagonal nanosheet on the Zn surface and (b, c) the corresponding EDS results. The actual ratio of Zn to S elements is 4.8, slightly higher than the theoretical value (4.0) for byproduct $Zn_4SO_4(OH)_6{\cdot}xH_2O$. This may be attributed to the deposition of surface-bound Zn metal.

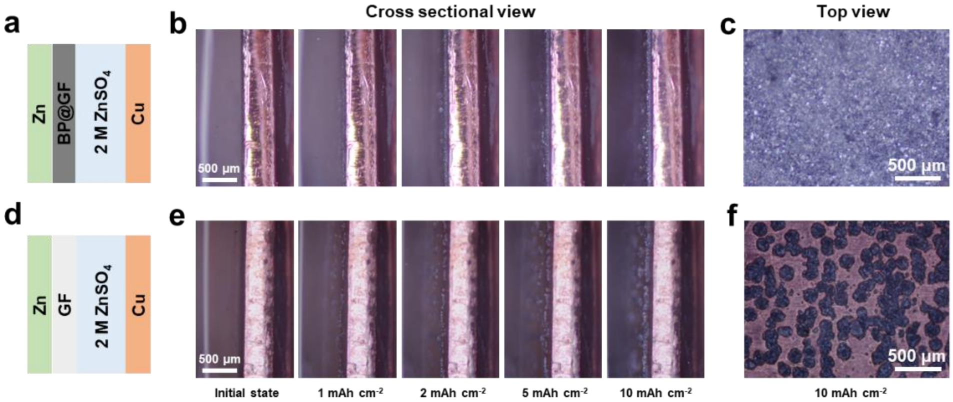

Fig. S7 In situ optical observations of Zn deposition on Cu foil with (a-c) BP@GF and (d-f) GF separators. The separators are tightly attached to the Zn anode.

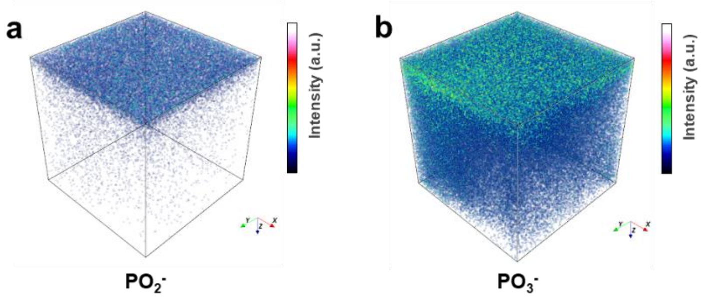

Fig. S8 3D views of the (a) PO_2^- and (b) PO_3^- signals by TOF-SIMS.

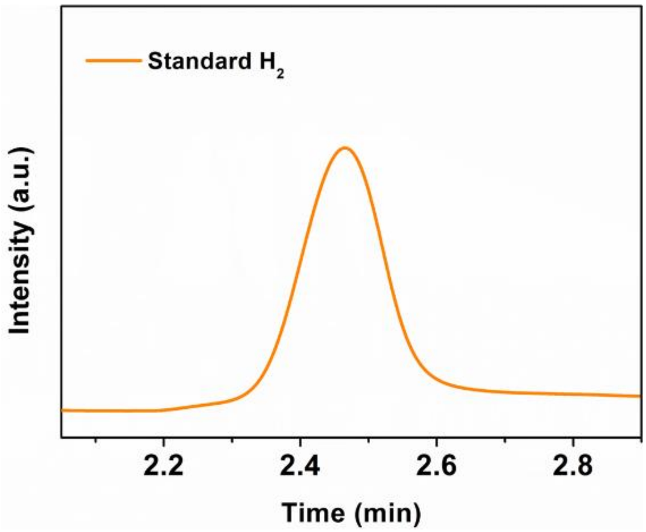

Fig. S9 Standard GC profile of hydrogen.

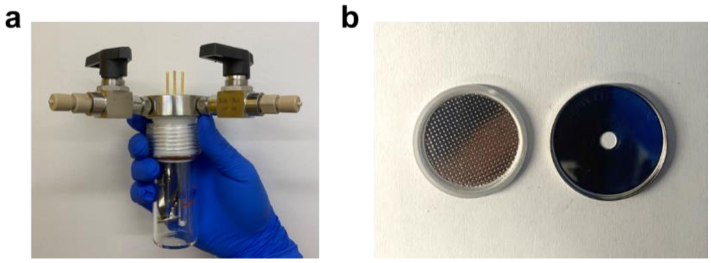

Fig. S10 (a) The container (20 mV) used for GC test, inside is a Zn||Zn symmetrical cells coin cell. (b) A hole (2 mm) is opened on the positive shell to release hydrogen.

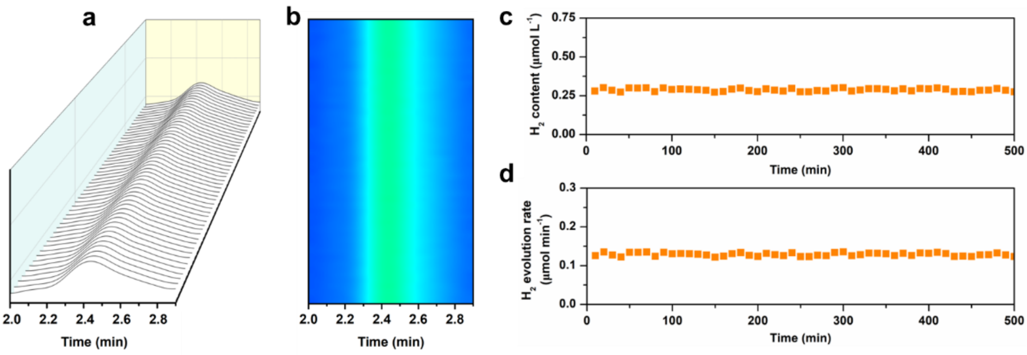

Fig. S11 (a) in situ electrochemical GC profiles and (b) the corresponding contour maps. (c) H_2 content and (d) H_2 evolution rate of ZPO@Zn||ZPO@Zn symmetrical cell with GF separator at 5 mA cm^{-2}.

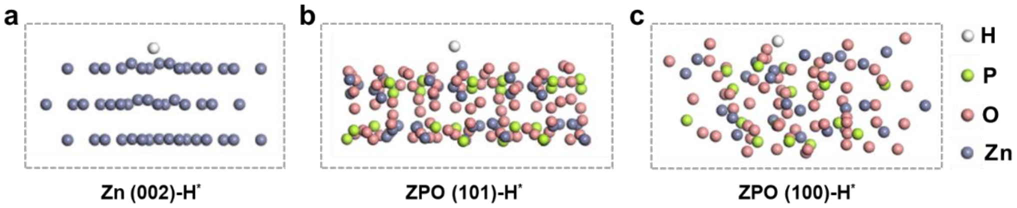

Fig. S12 Geometric models of (a) Zn (002)-H^*, (b) ZPO (101)-H^* and (c) ZPO (100)-H^* in DFT calculations.

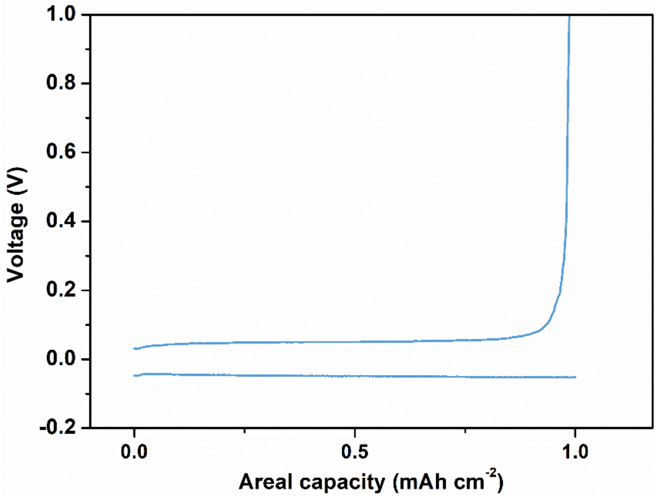

Fig. S13 GCD curve of BP@GF cell at the 1800th cycle.

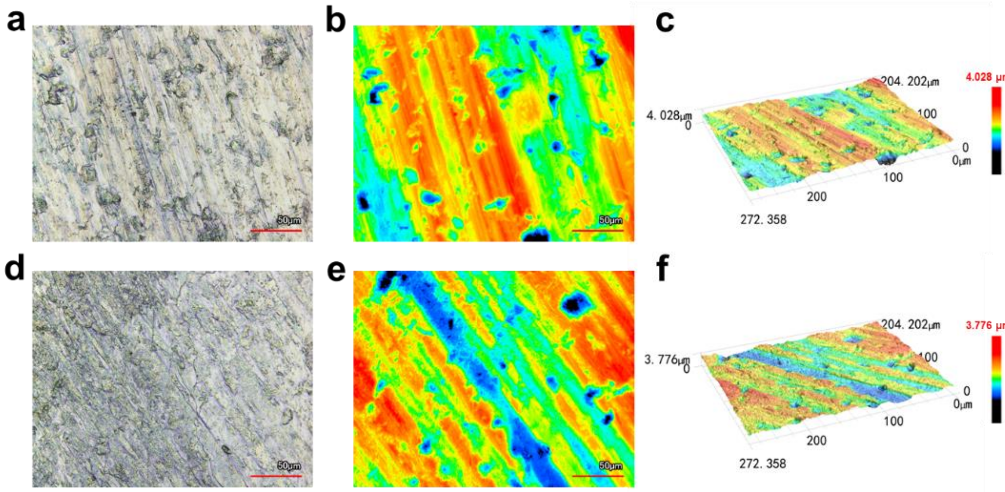

Fig. S14 (a, d) Top-view, (b, e) 2D contour map and (c, f) 3D view confocal images of the pristine Zn and the Zn foil after immersing in 20 mM zinc phosphate aqueous solution (ZPO@Zn), respectively.

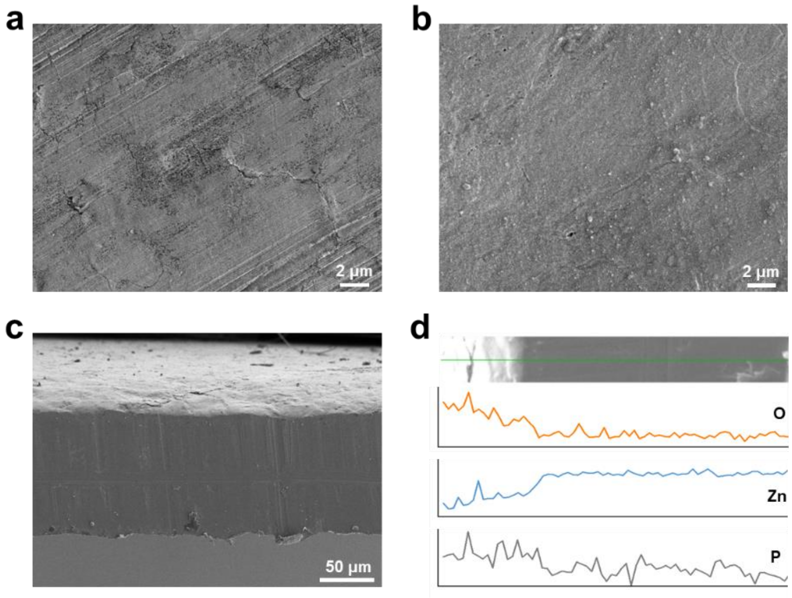

Fig. S15 Top view SEM images of (a) pristine Zn and (b) ZPO@Zn. (c) Cross sectional SEM images of ZPO@Zn and (d) the corresponding linear EDS results.

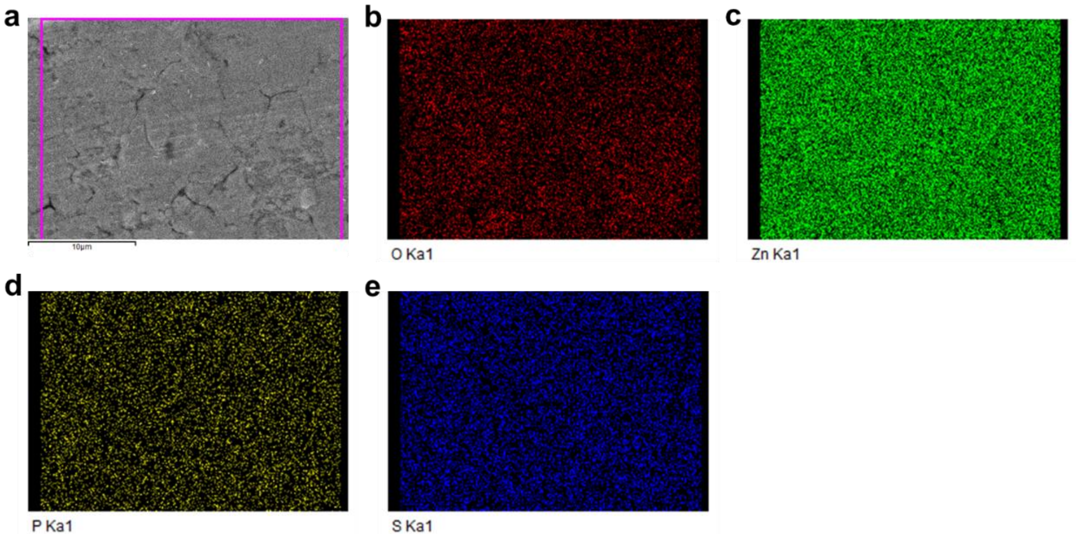

Fig. S16 Top view SEM image and the corresponding EDS mapping results of ZPO@Zn.

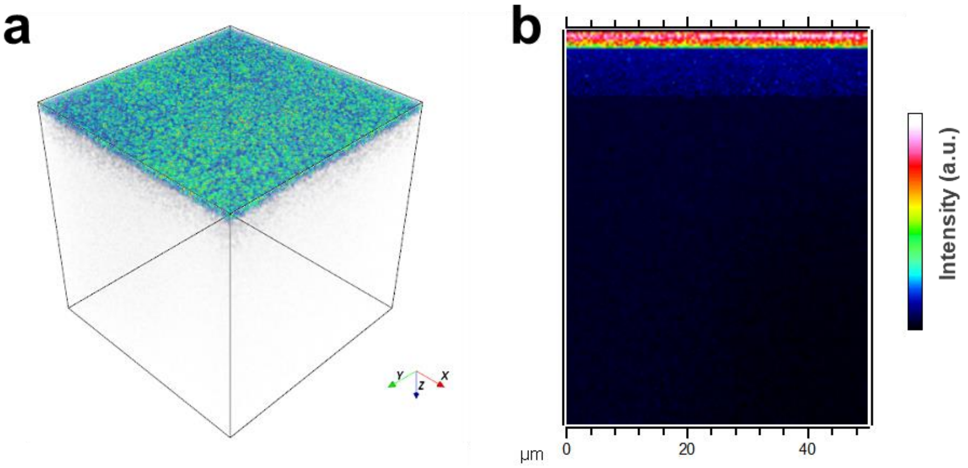

Fig. S17 (a) 3D view and (b) cross sectional view of PO_4^- signal revealed by TOF-SIMS for ZPO@Zn.

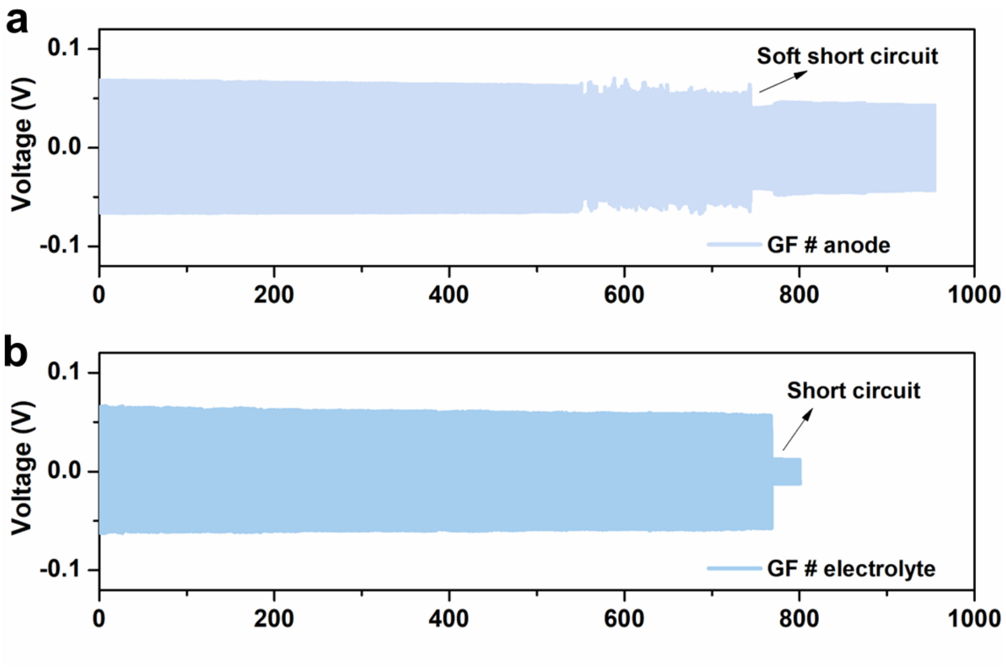

Fig. S18 Time-voltage curves of Zn||Zn symmetrical cells with GF#anode and GF#electrolyte at 5 mA cm^{-2} & 1 mAh cm^{-2}.

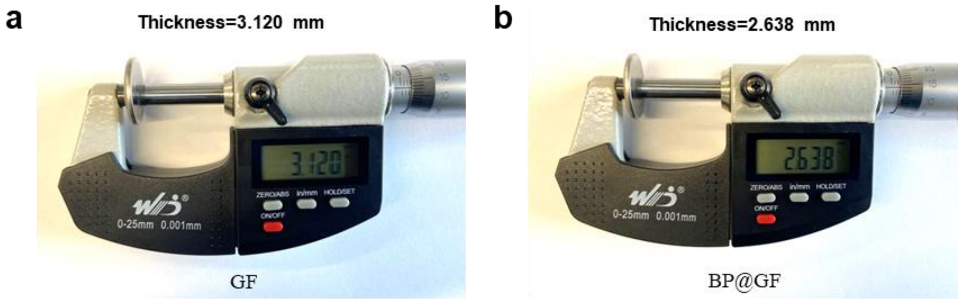

Fig. S19 The thicknesses of Zn||Zn symmetrical cells with (a) GF and (b) BP@GF separators after cycling 100 h at 50 mA cm^{-2} & 1 mAh cm^{-2}.

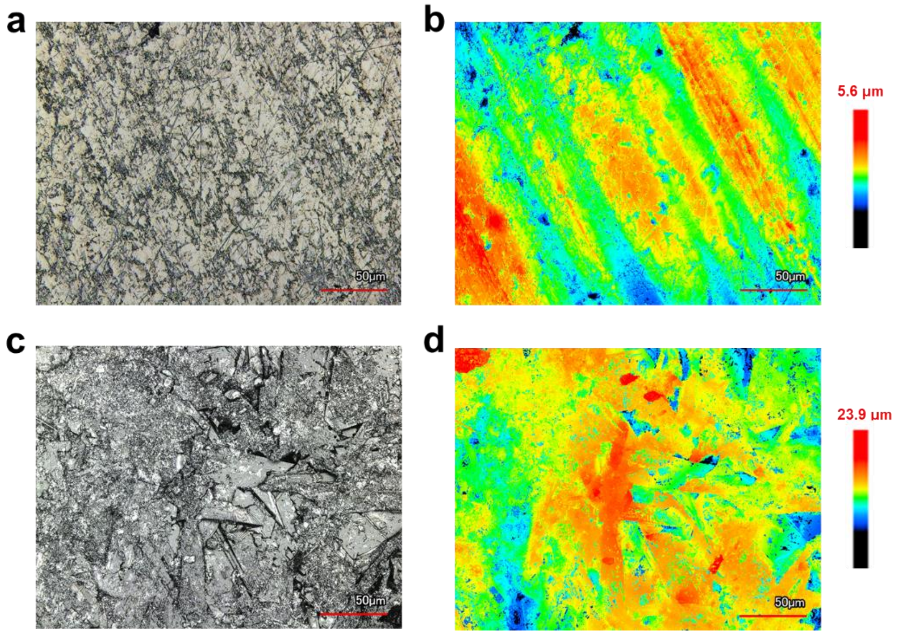

Fig. S20 (a, c) Top view and (b, d) 2D contour maps of confocal images of the cycled Zn (100 h) in the Zn||Zn symmetrical cells with BP@GF and GF separators at 5 mA cm^{-2} & 1 mAh cm^{-2}, respectively.

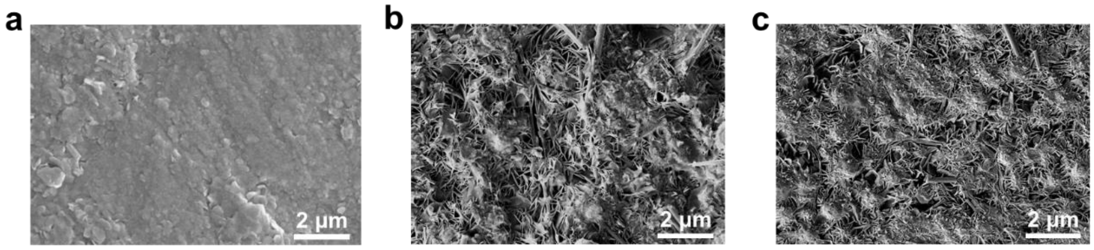

Fig. S21 Top view SEM mages of (a) Zn after cycling 300 h in Zn||Zn symmetrical cells with (a) BP@GF separator and 2 M $ZnSO_4$ electrolyte, (b) ZPO@Zn after cycling 300 h in ZPO@Zn||ZPO@Zn symmetrical cells with GF separator and 2 M $ZnSO_4$ electrolyte, (c) Zn after cycling 300 h in Zn||Zn symmetrical cells with GF separator and 20 mM H_3PO_4/2 M $ZnSO_4$ electrolyte.

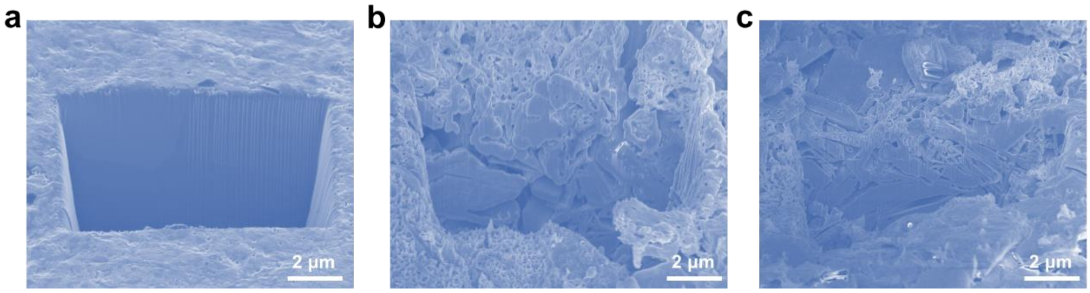

Fig. S22 Cross sectional images produced by FIB for (a) Zn after cycling 300 h in Zn||Zn symmetrical cells with (a) BP@GF separator and 2 M $ZnSO_4$ electrolyte, (b) ZPO@Zn after cycling 300 h in ZPO@Zn||ZPO@Zn symmetrical cells with GF separator and 2 M $ZnSO_4$ electrolyte, (c) Zn after cycling 300 h in Zn||Zn symmetrical cells with GF separator and 20 mM H_3PO_4/2 M $ZnSO_4$ electrolyte.

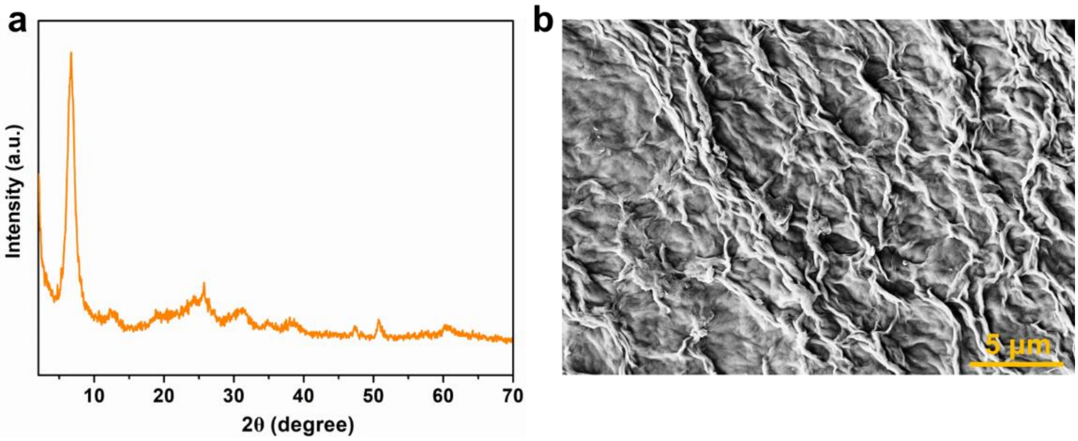

Fig. S23 (a) XRD pattern and (b) SEM image of $V_2O_5{\cdot}nH_2O$.

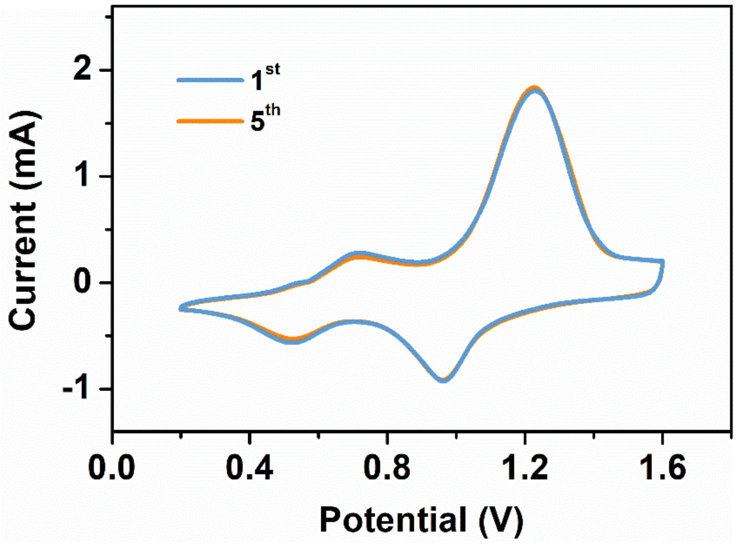

Fig. S24 CV curves of Zn||$V_2O_5 \cdot nH_2O$@FCP cells BP@GF separator.

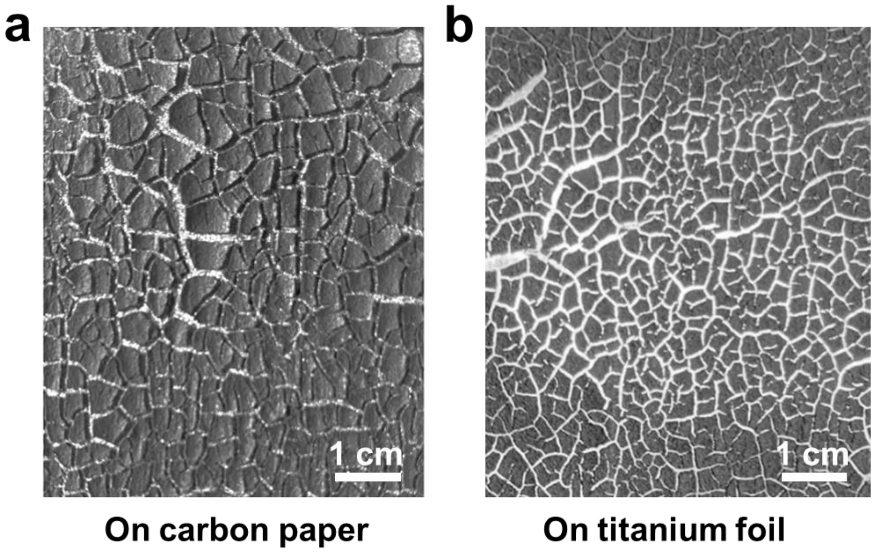

Fig. S25 $V_2O_5 \cdot nH_2O$ cathodes with high-loadings (>20 mg cm^{-2}) on traditional current collectors of (a) carbon paper and (b) titanium foil.

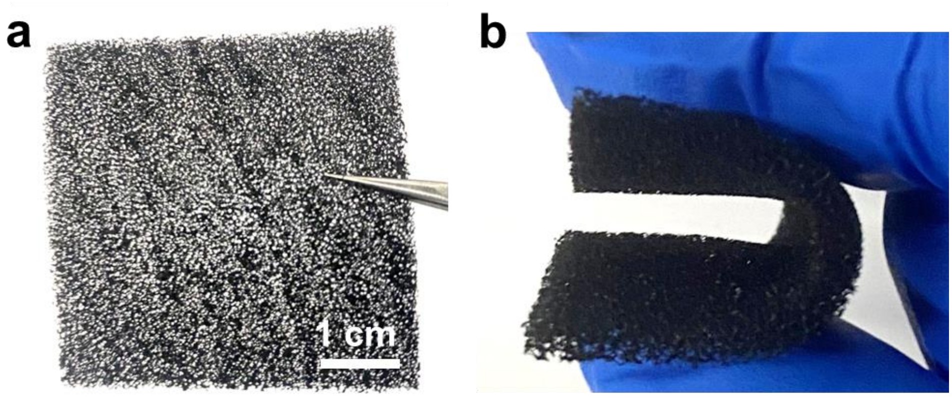

Fig. S26 (a) Optical photos of FCP current collector at (a) natural state and (b) bending state, highlighting the good flexibility.

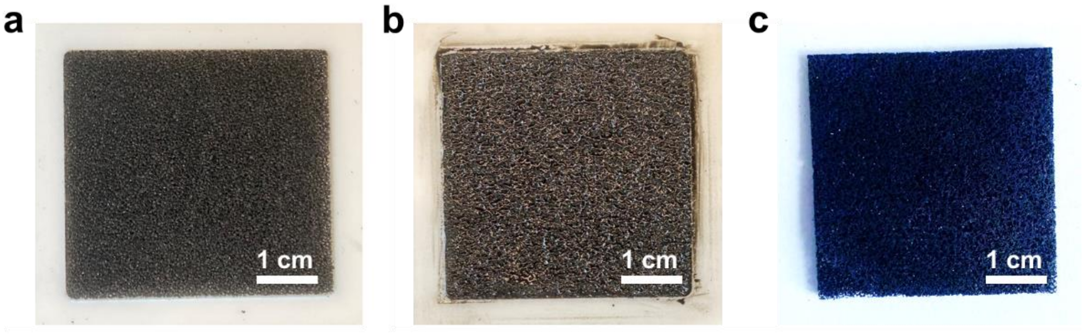

Fig. S27 Optical photos of manufacture of $V_2O_5 \cdot nH_2O$ cathode with high mass loadings. (a) PTFE groove filled with FCP. (b) After the slurry injection. (c) After drying.

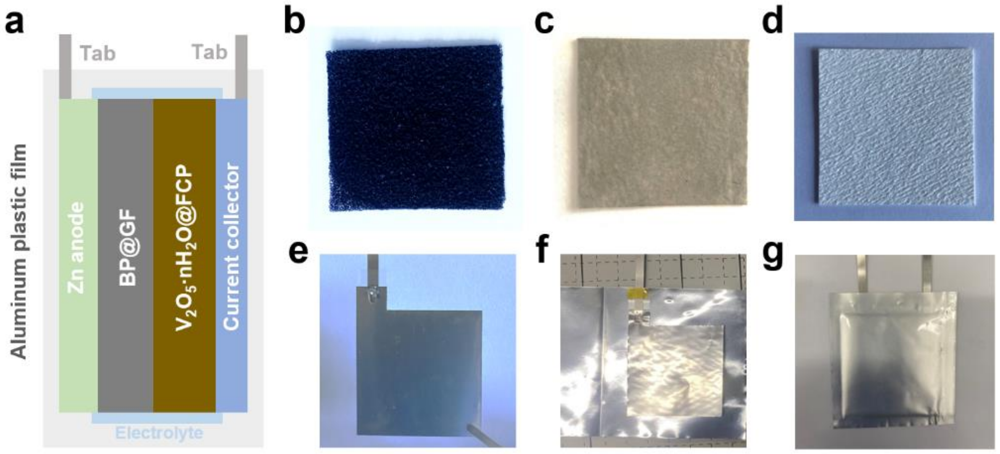

Fig. S28 (a) Schematic of pouch cell. Optical photos of (b) $V_2O_5 \cdot nH_2O$@FCP cathode, (c) BP@GF, (d) GF, (e) Zn foil (100 μm), (f) Ti foil and (g) pouch cell.

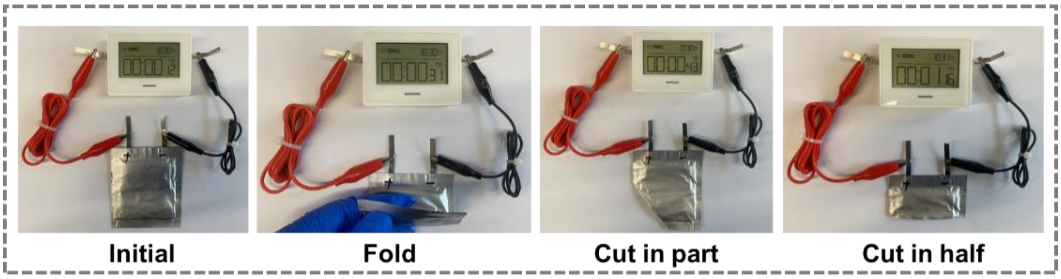

Fig. S29 Digital photos showing the pouch cells with BP@GF as the separator to power an electronic timer at different states, highlighting the reliability and safety.

Table S1 Comparison of the CPC and areal capacity in symmetrical Zn||Zn battery between our work and very recent reports.

Improvement methods	Current density (mA cm^{-2})	Areal capacity (mAh cm^{-2})	CPC (Ah cm^{-2})	Reference
4 m $Zn(OTF)_2$ + 0.5 m $Me_3EtNOTF$	0.5	0.25	1.5	[4]
4 m $Zn(BF_4)_2$/EG	0.5	0.25	1	[5]
$Zn(OTF)_2$ saturated 50vol%PC/H_2O	10	0.5	4	[6]
1 M $ZnSO_4$ in Betaine /H_2O (1:1 in weight)	0.5	0.5	1.06	[7]
1 M $ZnAc_2$ + 4 M NH_4I	1	0.5	0.3	[8]
$Zn_3(PO_4)_2{\cdot}4H_2O$ coated Zn	20	1	15.6	[9]
$BaTiO_3$/PVDF/GF separator	10	1	9.5	[10]
1 M $ZnSO_4$+25 mM $NH_4H_2PO_4$	10	1	4.65	[11]
2 M $ZnSO_4$ + 0.5 mg mL^{-1} C_3N_4QDs	5	1	1	[12]
2 M $ZnSO_4$ + 0.05 M SG	1	1	0.5	[13]
2 M $ZnSO_4$ + 50 mM DOTf	4	4	0.7	[14]
1 M $ZnSO_4$+ 0.5 wt% silk fibroin	10	5	2.5	[15]
2 M $Zn(NH_2SO_3)_2$	5	5	1.5	[16]
1 M $Zn(ClO_4)_2$ +10mM β-CD	5	5	0.875	[17]
2 M $ZnSO_4$ + 5vol% NMP	5	5	0.488	[18]
2 M $ZnSO_4$ + 1 vol% GBL	10	10	5.85	[19]
2 M $ZnSO_4$ + 0.02 M $BMIm^+$	10	10	5	[20]
2 M $ZnSO_4$+ 2 g/L $CeCl_3$	40	10	3.6	[21]
2 M $ZnSO_4$+ 0.5 g/L Saccharin	10	10	2.75	[22]
2 M $ZnSO_4$ + 5 vol% NMP	10	10	2.1	[23]
3 M $Zn(OTF)_2$ +20 mM $Zn(NO_3)_2$	10	10	0.45	[24]
BP@GF	5<br>50<br>50	1<br>1<br>10	8.75<br>50<br>12.5	This work

m: mol kg^{-1}

M: mol L^{-1}
$Zn(OTF)_2$: zinc trifluoromethanesulfonate
$Me_3EtNOTF$: trimethylethyl ammonium trifluoromethanesulfonate
EG: ethylene glycol
PC: propylene carbonate
C_3N_4QDs: graphitic carbon nitride quantum dots
SG: sodium glycerophosphate ($C_3H_7Na_2O_6P$)
DOTf: N, N-dimethylformamidium trifluoromethanesulfonate
β-CD: β-cyclodextrin
NMP: N-methyl-2-pyrrolidone
GBL: gamma butyrolactone
$BMIm^+$: 1-butyl-3-methylimidazolium cation

Table S2 Comparison of the CPC and areal capacity in full battery between our work and very recent reports.

	Cathode	Mass loadings (mg cm^{-2})	Areal capacity (mAh cm^{-2})	CPC (Ah cm^{-2})	Reference
Coin cell	$Zn_{0.25}V_2O_5$	4	0.8	0.8	[14]
	PANI	1.5	0.18	0.25	[13]
	V_6O_{13}	4.1	1.07	2.14	[25]
	V_2O_5	6.4	1.92	0.38	[26]
	MnO_2	6.3	1.95	0.8	[27]
	$VOPO_4$	2	0.2	0.6	[12]
	This work	34.1	7.36	2.8	
Pouch cell	V_2O_5	12	2.06	0.21	[28]
	$NaV_3O_8 \cdot 1.5H_2O$	3	0.35	0.52	[29]
	$VOPO_4$	3	0.36	0.04	[4]
	AlVO-DMF	10	2.84	0.09	[30]
	This work	28.7	5.3	0.88	

PANI: polyaniline

AlVO-DMF: $Al_xV_2O_5$-N,N-dimethylformamide

References:

[1] a) J. Li, Q. Lin, Z. Zheng, L. Cao, W. Lv, Y. Chen, *ACS Appl. Mater. Interfaces* **2022**, 14, 12323; b) J. Yang, H. Hua, H. Yang, P. Lai, M. Zhang, Z. Lv, Z. Wen, C. C. Li, J. Zhao, Y. Yang, *Adv. Energy Mater.* **2023**, 13, 2204005.

[2] J. P. Perdew, K. Burke, M. Ernzerhof, *Phys. Rev. Lett.* **1996**, 77, 3865.

[3] a) Y. Zheng, Y. Jiao, Y. Zhu, L. H. Li, Y. Han, Y. Chen, A. Du, M. Jaroniec, S. Z. Qiao, *Nat. Commun.* **2014**, 5, 1; b) M. Qu, Y. Jiang, M. Yang, S. Liu, Q. Guo, W. Shen, M. Li, R. He, *Applied Catalysis B: Environmental* **2020**, 263, 118324.

[4] L. Cao, D. Li, T. Pollard, T. Deng, B. Zhang, C. Yang, L. Chen, J. Vatamanu, E. Hu, M. J. Hourwitz, *Nat. Nanotechnol.* **2021**, 16, 902.

[5] D. Han, C. Cui, K. Zhang, Z. Wang, J. Gao, Y. Guo, Z. Zhang, S. Wu, L. Yin, Z. Weng, *Nat. Sustain.* **2022**, 5, 205.

[6] F. Ming, Y. Zhu, G. Huang, A.-H. Emwas, H. Liang, Y. Cui, H. N. Alshareef, *J. Am. Chem. Soc.* **2022**, 144, 7160.

[7] H. Ren, S. Li, B. Wang, Y. Zhang, T. Wang, Q. Lv, X. Zhang, L. Wang, X. Han, F. Jin, *Adv. Mater.* **2023**, 35, 2208237.

[8] Q. Zhang, Y. Ma, Y. Lu, Y. Ni, L. Lin, Z. Hao, Z. Yan, Q. Zhao, J. Chen, *J. Am. Chem. Soc.* **2022**, 144, 18435.

[9] H. J. Kim, S. Kim, K. Heo, J. H. Lim, H. Yashiro, S. T. Myung, *Adv. Energy Mater.* **2023**, 13, 2203189.

[10] Y. Liang, D. Ma, N. Zhao, Y. Wang, M. Yang, J. Ruan, G. Yang, H. Mi, C. He, P. Zhang, *Adv. Funct. Mater.* **2022**, 32, 2112936.

[11] W. Zhang, Y. Dai, R. Chen, Z. Xu, J. Li, W. Zong, H. Li, Z. Li, Z. Zhang, J. Zhu, *Angew. Chem. Int. Ed.* **2023**, 62, e202212695.

[12] W. Zhang, M. Dong, K. Jiang, D. Yang, X. Tan, S. Zhai, R. Feng, N. Chen, G. King, H. Zhang, *Nat. Commun.* **2022**, 13, 5348.

[13] J. Hao, L. Yuan, Y. Zhu, M. Jaroniec, S. Z. Qiao, *Adv. Mater.* **2022**, 34, 2206963.

[14] C. Li, A. Shyamsunder, A. G. Hoane, D. M. Long, C. Y. Kwok, P. G. Kotula, K. R. Zavadil, A. A. Gewirth, L. F. Nazar, *Joule* **2022**, 6, 1103.

[15] J. Xu, W. Lv, W. Yang, Y. Jin, Q. Jin, B. Sun, Z. Zhang, T. Wang, L. Zheng, X. Shi, *ACS Nano* **2022**, 16, 11392.

[16] X. Xu, H. Su, J. Zhang, Y. Zhong, Y. Xu, Z. Qiu, H. B. Wu, X. Wang, C. Gu, J. Tu, *ACS Energy Lett.* **2022**, 7, 4459.

[17] M. Qiu, P. Sun, Y. Wang, L. Ma, C. Zhi, W. Mai, *Angew. Chem. Int. Ed.* **2022**, 61, e202210979.

[18] T. C. Li, Y. Lim, X. L. Li, S. Luo, C. Lin, D. Fang, S. Xia, Y. Wang, H. Y. Yang, *Adv. Energy Mater.* **2022**, 12, 2103231.

[19] H. Huang, D. Xie, J. Zhao, P. Rao, W. M. Choi, K. Davey, J. Mao, *Adv. Energy Mater.* **2022**, 12, 2202419.

[20] H. Zhang, Y. Zhong, J. Li, Y. Liao, J. Zeng, Y. Shen, L. Yuan, Z. Li, Y. Huang, *Adv. Energy Mater.* **2023**, 13, 2203254.

[21] Z. Hu, F. Zhang, Y. Zhao, H. Wang, Y. Huang, F. Wu, R. Chen, L. Li, *Adv. Mater.* **2022**, 34, 2203104.

[22] C. Huang, X. Zhao, S. Liu, Y. Hao, Q. Tang, A. Hu, Z. Liu, X. Chen, *Adv. Mater.* **2021**, 33, 2100445.

[23] D. Wang, D. Lv, H. Liu, S. Zhang, C. Wang, C. Wang, J. Yang, Y. Qian, *Angew. Chem.* **2022**, 134, e202212839.

[24] D. Li, L. Cao, T. Deng, S. Liu, C. Wang, *Angew. Chem. Int. Ed.* **2021**, 60, 13035.

[25] Q. Yang, L. Li, T. Hussain, D. Wang, L. Hui, Y. Guo, G. Liang, X. Li, Z. Chen, Z. Huang, *Angew. Chem.* **2022**, 134, e202112304.

[26] K. Zhao, G. Fan, J. Liu, F. Liu, J. Li, X. Zhou, Y. Ni, M. Yu, Y.-M. Zhang, H. Su, *J. Am. Chem. Soc.* **2022**, 144, 11129.

[27] R. Wang, S. Xin, D. Chao, Z. Liu, J. Wan, P. Xiong, Q. Luo, K. Hua, J. Hao, C. Zhang, *Adv. Funct. Mater.* **2022**, 2207751.

[28] R. Guo, X. Liu, F. Xia, Y. Jiang, H. Zhang, M. Huang, C. Niu, J. Wu, Y. Zhao, X. Wang, *Adv. Mater.* **2022**, 34, 2202188.

[29] Y. Lv, M. Zhao, Y. Du, Y. Kang, Y. Xiao, S. Chen, *Energy Environ. Sci.* **2022**, 15, 4748.

[30] F. Wan, Z. Hao, S. Wang, Y. Ni, J. Zhu, Z. Tie, S. Bi, Z. Niu, J. Chen, *Adv. Mater.* **2021**, 33, 2102701.
